# Characterization of two different alginate-based bioinks and the influence of melanoma growth within

**DOI:** 10.1038/s41598-024-63642-3

**Published:** 2024-06-05

**Authors:** Raphael Schipka, Stefanie Heltmann-Meyer, Dominik Schneidereit, Oliver Friedrich, Jonas Röder, Aldo R. Boccaccini, Stefan Schrüfer, Dirk W. Schubert, Raymund E. Horch, Anja K. Bosserhoff, Andreas Arkudas, Annika Kengelbach-Weigand, Rafael Schmid

**Affiliations:** 1 Department of Plastic and Hand Surgery, Laboratory for Tissue Engineering and Regenerative Medicine, University Hospital Erlangen, Friedrich-Alexander-University Erlangen-Nürnberg, 91054 Erlangen, Germany; 2 Institute of Biomaterials, Friedrich-Alexander-University Erlangen-Nürnberg, 91058 Erlangen, Germany; 3 Institute of Medical Biotechnology, Department of Chemical and Biological Engineering, Friedrich-Alexander-University Erlangen-Nürnberg, 91052 Erlangen, Germany; 4 Institute of Polymer Materials, Friedrich-Alexander-University Erlangen-Nürnberg, 91058 Erlangen, Germany; 5 RevoBITs, Friedrich-Alexander-University Erlangen-Nürnberg, 91058 Erlangen, Germany; 6 Institute of Biochemistry, Friedrich-Alexander-University Erlangen-Nürnberg, 91054 Erlangen, Germany

**Keywords:** Hydrogel, Stiffness, Alginate, Melanoma, 3D-printing, Biomaterials - cells, Biomedical engineering, Tissue engineering

## Abstract

Extrusion-based bioprinting is an established method in biofabrication. Suitable bioinks have fundamentally different compositions and characteristics, which should be examined, in order to find a perfect model system. Here, we investigate the effect of two alginate-based, yet unalike 3D-printed bioinks, pre-crosslinked alginate-dialdehyde gelatin (ADA-GEL) and a mixture of alginate, hyaluronic acid, and gelatin (Alg/HA/Gel), on the melanoma cell line Mel Im and vice versa in terms of stiffness, shrinkage, cellular behavior and colony formation over 15 days. Rheological stiffness measurements revealed two soft gels with similar storage moduli. The cells did not have a significant impact on the overall stiffness, whereas ADA-GEL (2.5/2.5%) was significantly stiffer than Alg/HA/Gel (0.5/0.1/3%). Regarding the shrinkage of printed constructs, cells had a significant influence, especially in ADA-GEL, which has covalent bonds between the oxidized alginate and gelatin. Multi-photon microscopy exhibited proliferation, cell spreading and migration in ADA-GEL with cell–cell and cell–matrix interaction, dissimilarly to Alg/HA/Gel, in which cells formed spherical, encapsulated colonies. Scanning electron microscopy and histology showed degradation and multi-layered growth on ADA-GEL and fewer examples of escaped cells on Alg/HA/Gel. Both gels serve as proliferation bioink for melanoma with more necrosis in deeper Alg/HA/Gel colonies and differences in spreading and matrix interaction. These findings show the importance of proper characterization of the bioinks for different applications.

## Introduction

3D printing has so far been established in various areas, initially in the industry and subsequently also in areas relevant to medicine. Here, shapes directly adapted for the patient’s individual treatment, such as 3D-printed prostheses or orthoses, were produced. This customization offers the possibility to make the treatment more comfortable for the patient, to improve it and ultimately shorten the duration of healing ^1^.

However, it is not only solid materials that can be shaped using 3D printing; there is an increasing number of applications in regenerative medicine. The most prominent example being tissue engineering (TE), a combination of engineering and biosciences to create artificial tissue. For this purpose, the techniques in science and research must also be adapted in order to come as close as possible to the in vivo conditions in the patient. In the past, models were predominantly 2D models in cell culture. Nevertheless, there are limitations in terms of cell–cell and cell–matrix interactions, which do not correspond to the 3D environment ^2,3^. 3D models can be improved in terms of spatial arrangement and reproducibility using 3D printing processes ^4,5^ which can also be of use in cancer research ^3,6^. The cells are encapsulated in a microenvironment that comes as close as possible to the tumor environment in vivo and can be examined more closely with regard to tumor behavior, such as growth, spread and metastasis ^7–10^.

Various tumor cell types can be examined, such as malignant melanoma. Melanoma is a form of skin cancer and its cutaneous form is responsible for a large portion (75%) of deaths associated with skin cancer ^11^. Melanomas, characterized by their highly metastatic and malignant nature, serve as a representative paradigm for tumors influenced by various intrinsic and extrinsic factors throughout their development ^12^. They are caused by malignant changes in the pigment-producing melanocytes which are found throughout the body, particularly in the basal cell layer of the epidermis and in the hair follicles. In tumors, strong heterogenicity of the tumor cells can be observed, e. g. the tumor cells behave differently depending on their position relative to the surface. Cells that are located on or near the surface of the tumor and, therefore, have a good supply of nutrients and oxygen can multiply, while cells that are located inside the tumor turn into a non-dividing stage. In larger tumors, necrosis of the tumor cells in the center can also occur ^13^. These phenomena can be mimicked in 3D in vitro models ^14^.

Bioinks are suitable for replicating the in vivo environment as accurately as possible ^15^. These inks contain cells and hydrogels which consist largely of water and can be adapted to the tumor environment in terms of their composition, stiffness and diffusion properties thorough alterations of their composition. Natural materials usually offer good biocompatibility and are non-toxic. Alginate is a polysaccharide from brown algae. It is already of use in medicine and the food industry as well as in biofabrication ^16–18^. Alginate can be covalently bound to gelatin (GEL) by modification using partial oxidation, resulting in the so-called alginate-dialdehyde gelatin (ADA-GEL). The coupled gelatin in turn provides integrin-binding sequences (RGD) to which cells can attach better, thus promoting biocompatibility and angiogenesis. Gelatin can also be used as a component of a hydrogel. Gelatin is made from various types of hydrolyzed collagen which is easily degradable and does not cause severe immune reactions, as can be the case with non-hydrolyzed collagen ^19,20^. It is a component of the extracellular matrix (ECM) and can, therefore, also be used to reproduce it. Another typical component is hyaluronic acid which plays a major role in cell proliferation, migration and differentiation^21^.

In this study, a pre-crosslinked ADA-GEL ink already known in TE and Alg/HA/Gel, a three-component ink consisting of alginate, hyaluronic acid, and gelatin, were compared in terms of physical properties, degradation, and biological behavior in vitro. Both gels can be cross-linked using CaCl_2_. It has already been shown in both inks used here that they have good printability and also allow cell growth, both in vitro (endothelial cells and tumor cells) and in vivo (tumor cells), although endothelial cells do not survive in Alg/HA/Gel. In this paper melanoma cells were encapsulated in both gels, and a disk shape with a diameter of 20 mm and a height of 2 mm was printed using a 3D printer to gain a more detailed view on time-dependent changes. The constructs were then examined in a rheometer and compared with and without cells over 14 days. The behavior of the cells was microscopically observed using cytoskeletal staining, cell proliferation and cell cycle analysis using FUCCI transfected cells and examined via a multi-photon microscope over a period of 15 days to get a better insight into the behavior of the cells in the gel. Furthermore, a 3D-printed grid structure was examined histologically and via scanning electron microscopy.

## Results

### Characterization of printing and material parameters

**Figure 1:**
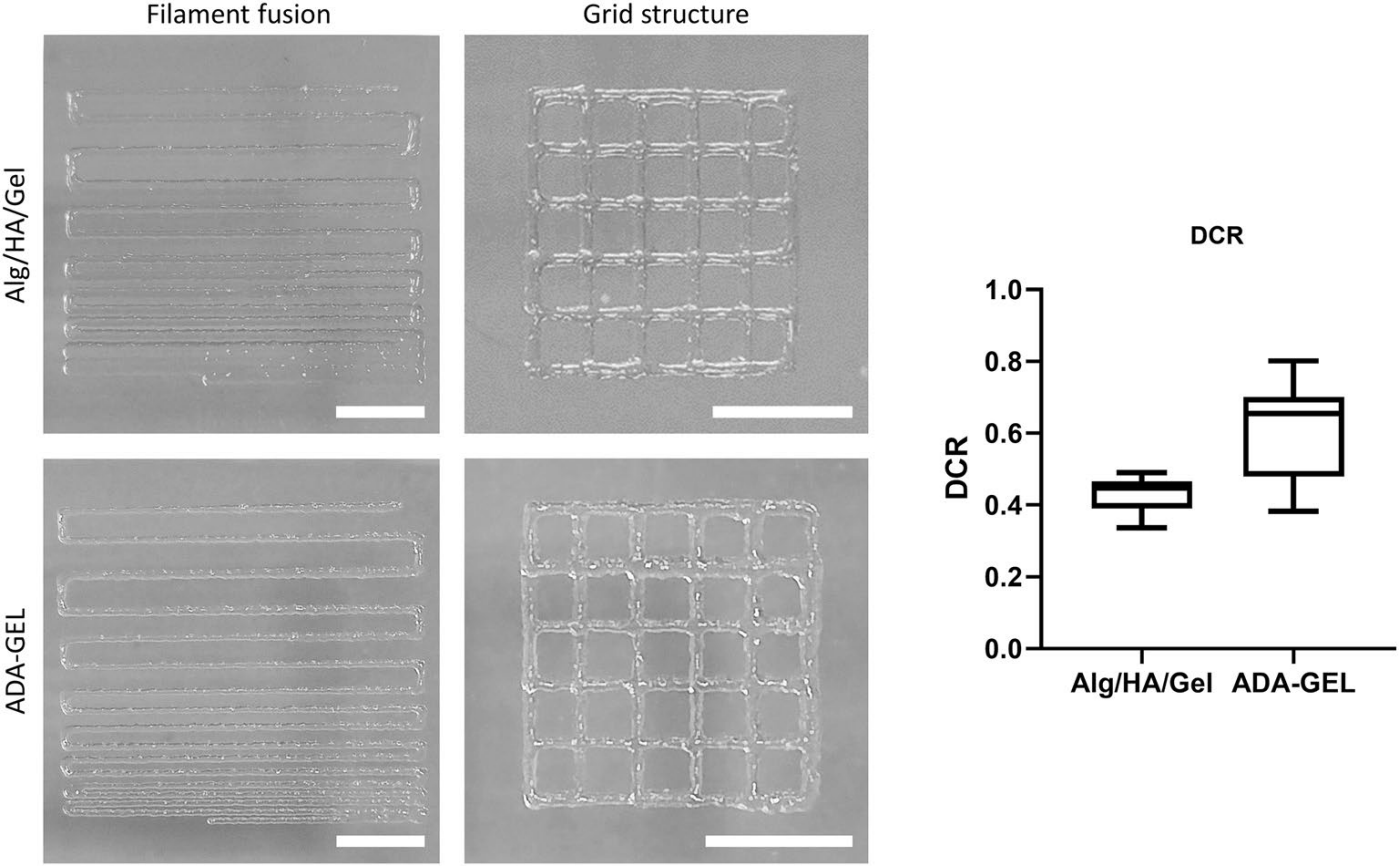
Printing test and DCR analysis of Alg/HA/Gel and ADA-GEL. The printing test of both inks with a filament fusion and a grid structure. Scale bar = 5 mm. DCR analysis of the overlapping strands in the grid structure. Data shown as box plot of technical replicates.

In the printing test, both inks used proved to have good printability. Specifically, this was demonstrated in the filament fusion test by the fact that both inks did not show any fusion of the print strands until a distance of less than 0.75 mm was reached (Fig. 1). This means that both inks can be classified as category B inks. With both hydrogels, correspondingly good grid structures could be printed. The evaluation of the diagonals of intersecting strands showed a diagonal cross ratio (DCR) value of 0.43 ± 0.05 for Alg/HA/Gel and 0.60 ± 0.14 for ADA-GEL (Fig. 1). This confirms that ADA-GEL has slightly better shape fidelity.

**Figure 2:**
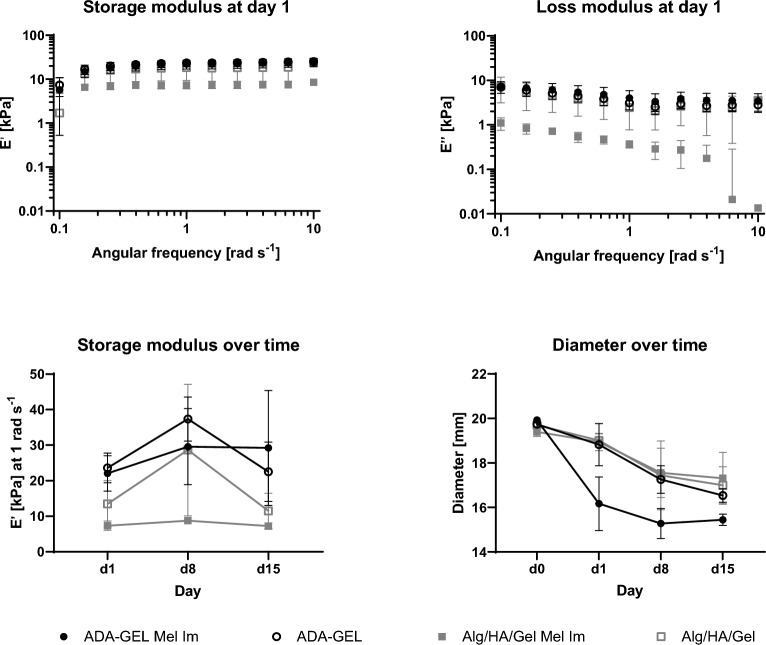
Rheological stiffness measurement and shrinkage behavior of Alg/HA/Gel and ADA-GEL with and without Mel Im cells. The examined gels showed mainly elastic behavior when comparing the storage and the loss moduli at day 1 (top). The storage modulus at 1 rad s^−1^ (bottom left) and the shrinkage of the samples (bottom right) over 15 days. With a significant change of the storage modulus at 1 rad s^−1^ for Alg/HA/Gel without cells from day 1 to day 8 and day 8 to day 15. As well as a significant change of the diameter of ADA-GEL with Mel Im from day 0 to day 1.

The behavior of the gels with and without cells was examined in more detail, including a rheological analysis of the inks’ stiffness and monitoring of the shrinkage behavior over time (Fig. 2).

A rheometer was used to measure the changes in storage (E′) and loss modulus (E″) in the two crosslinked inks Alg/HA/Gel (0.5% alginate, 0.1% HA, 3% gelatin) and pre-crosslinked ADA-GEL (2.5% ADA, 2.5% gelatin). The evaluation shows that the storage modulus differed from the loss modulus in both inks. The storage modulus was higher than the loss modulus, which indicates an elastic behavior of the inks. The three-way ANOVA showed a significant source of variation with respect to the gel and the day but not the cells.

Over time, the storage modulus at 1 rad s^−1^ differed between the inks. For Alg/HA/Gel with Mel Im cells, it was continuously below 10 kPa and stable over 15 days. Without cells, the storage modulus changed statistically significant from day 1 to day 8 and from day 8 to day 15, with a peak at 28.64 ± 18.52 kPa on day 8, this value being more than twice as high than on day 1 and day 15. ADA-GEL showed a similar behavior without cells. Specifically the values of the storage modulus on day 1 and 8 were very similar, but 1.6 times higher on day 8 (n.s.). With cells, the storage modulus increased from day 1 to day 8 and then remained at this level until day 15. Overall, the values of the Alg/HA/Gel ink were lower than those of ADA-GEL, which applied when comparing the gels with cells as well as those without.

Looking at the diameter of the samples, all of them shrank over time. The three-way ANOVA confirmed the gel, the cells, and the day as significant source of variation. On day 0, all samples had a very similar diameter (19.40 ± 0.20 mm to 19.93 ± 0.06 mm). ADA-GEL with Mel Im cells became significantly smaller on day 1 (19% shrinkage rate), but then remained at this level until day 15, shrinking by a total of 23%. The other gels had a similar behavior among them and showed the highest and significant shrinkage rate between day 1 and day 8. Compared to day 0, the diameter decreased by 16% (ADA-GEL), 11% (Alg/HA/Gel with Mel Im cells) and 14% (Alg/HA/Gel) over 15 days. Stiffness and diameter of the samples non-significantly correlated with a Spearman correlation coefficient r of -0.49.

### Cell behavior in both bioinks

**Figure 3:**
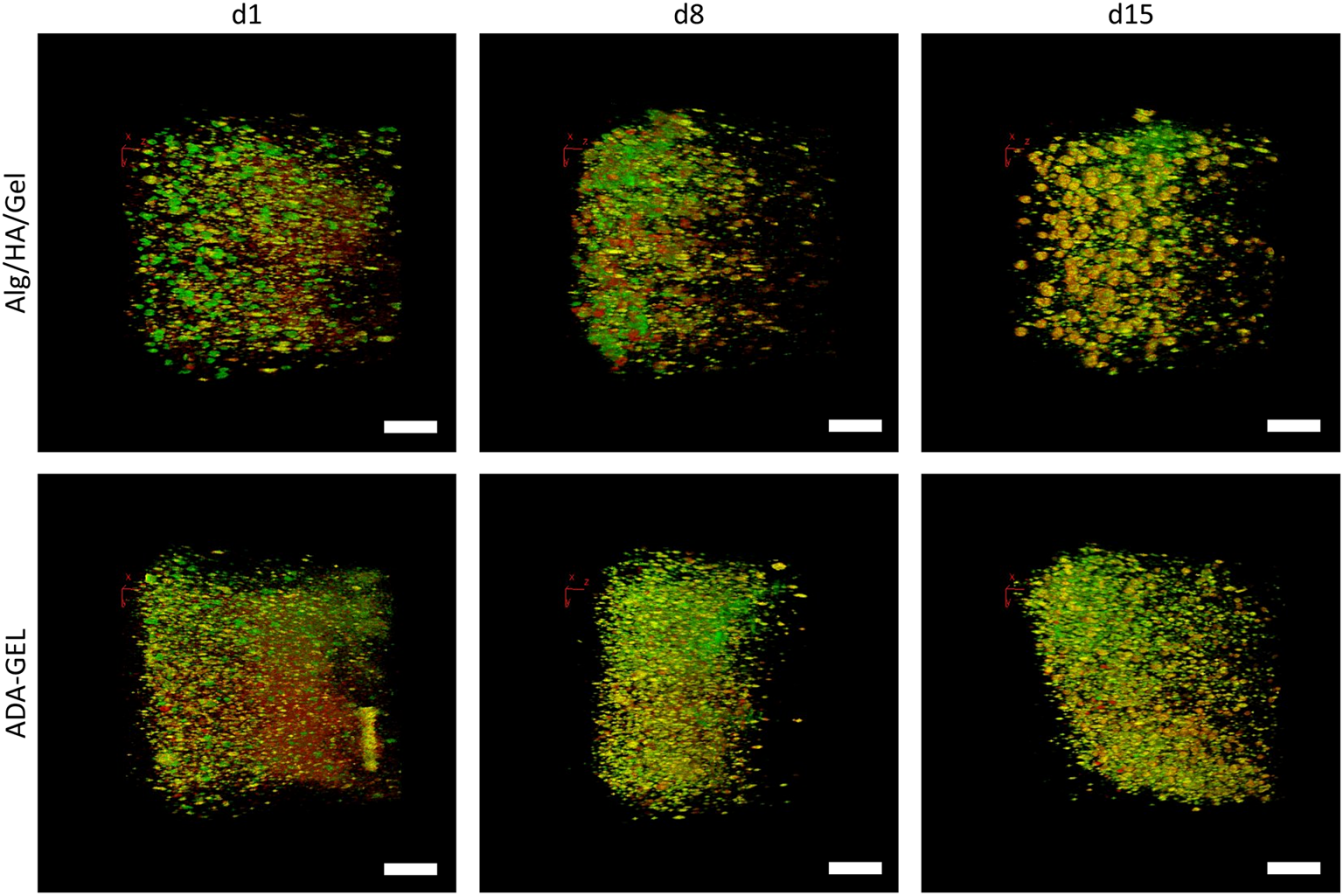
Cell cycle signal of Mel Im FUCCI in Alg/HA/Gel and ADA-GEL over 15 days. FUCCI signal of Mel Im cells on day 1, 8 and 15 in Alg/HA/Gel and ADA-GEL. On the left side of each image is the surface of the sample, facing into the sample to the right. In Alg/HA/Gel, cells growing in colonies and migrating closer to the gel surface were seen. In contrast, the cells in ADA-GEL tend to grow in smaller clusters and deeper into the gel. Green = live cells, red = dead cells; Scale bar = 200 μm.

**Figure 4:**
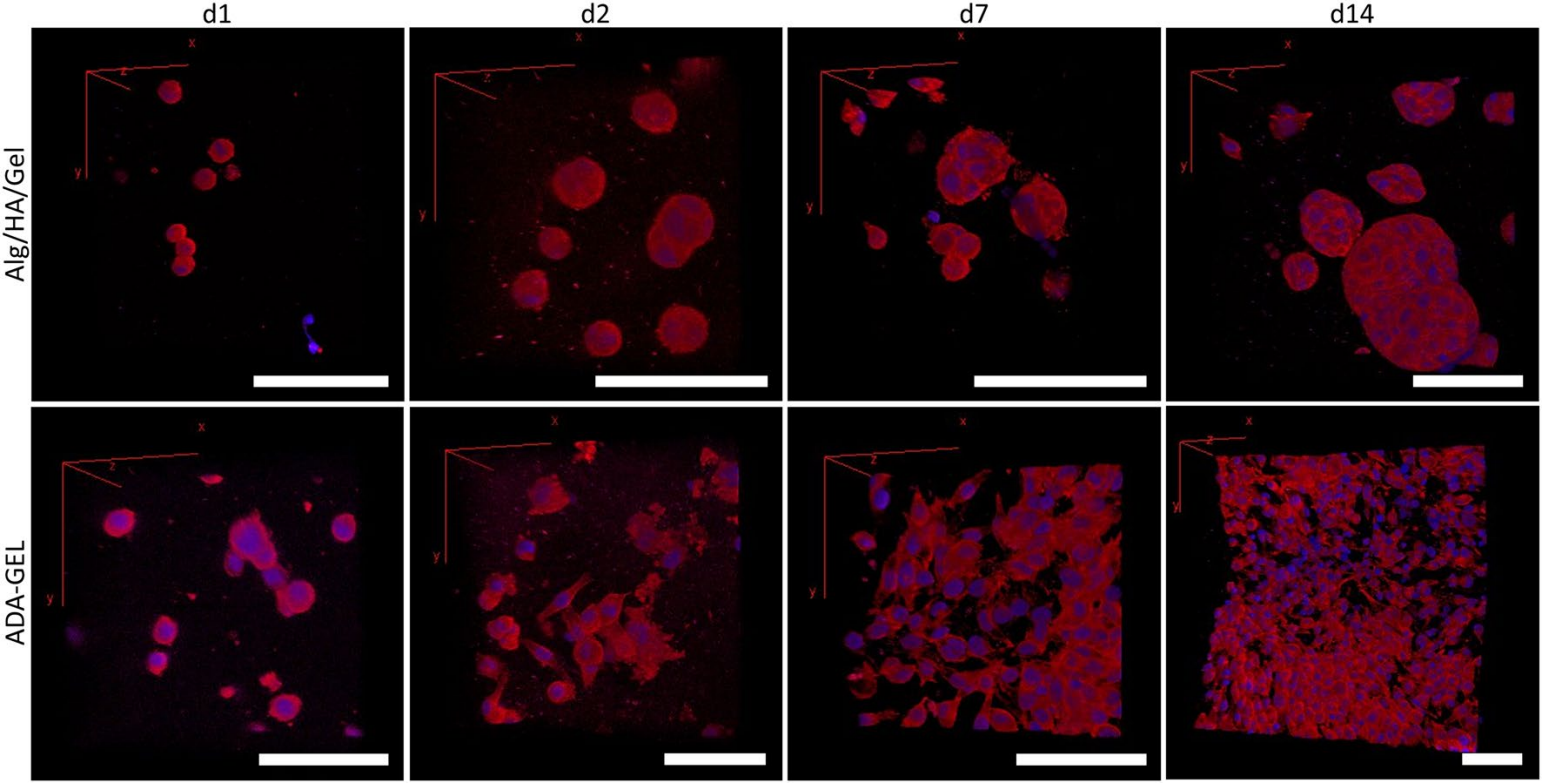
Alexa 635/DAPI staining of Mel Im in Alg/HA/Gel and ADA-GEL over 14 days. Morphological staining of Mel Im cells on day 1, 2, 7 and 14 in Alg/HA/Gel and ADA-GEL. In Alg/HA/Gel, spherical colonies formed over time; in ADA-GEL cells spread and interconnected networks were formed. Red = cytoskeleton, blue = nucleus; Scale bar = 30 μm.

**Figure 5:**
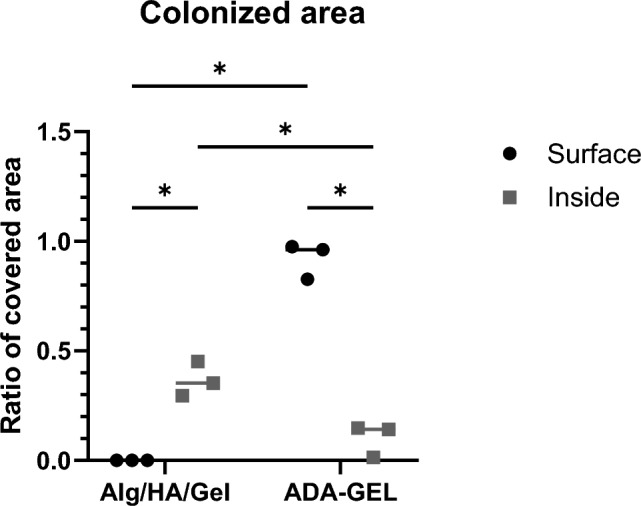
Quantification of the colonized area on day 14: Cells in Alg/HA/Gel grow in larger colonies within the gel while cells in ADA-GEL migrate and cover the surface. Data shown as mean and individual values of biological replicates. *p ≤ 0.05 (Shapiro–Wilk normality test followed by paired two-way ANOVA and Fisher’s LSD test).

The behavior of the cells in the inks was analyzed using multi-photon microscopy (MPM). First, the FUCCI signal (Fluorescent Ubiquitination-based Cell Cycle Indicator) of the cells was observed through the gel (Fig. 3), and the morphology of the cells was investigated using a Phalloidin Alexa 635/DAPI staining (Fig. 4), which was quantified (Fig. 5).

Living cells were found in both gels over a period of 15 days. They differed in their spatial distribution within the gel and in their clustering behavior. In Alg/HA/Gel, the cells were distributed throughout the deeper layers of the gel on day 1 but began to form smaller clusters after 24 h. In further progression, there were more and more cells close to the surface and fewer cells distant from it. Also, larger conglomerates of cells were observed. The clusters predominantly emitted in the green channel (S phase, G2 phase and M phase) on day 1. On day 8, clusters of green as well as red (G1 phase) cells were found. The clusters showed more of a superposition of green and red by day 15. In comparison, the cells in ADA-GEL behaved differently. Here, the gel was also interspersed with cells on day 1, but larger cell aggregates or clusters were rare, even observed over time. On the following days, it can be observed that the number of cells increases, but they continue to appear as single cells in the gel.Those cells are more frequently found near the surface and become fewer towards the inner part of the gel. The FUCCI signal mainly showed cells that were visible in the green channel, but a few red and yellow cells can also be found, which result from a superposition of both signals.

The cytoskeletal staining showed isolated cells in the gel on the day of printing in both gels. In the Alg/HA/Gel ink, as in the FUCCI analysis, from day 2, it was already shown that the cells formed clusters and proliferated within them. This means that the cell agglomerates became larger over time. The individual cells and their cytoskeleton could hardly be distinguished from each other, and the blue nuclei surrounded by a red cytoskeleton cloud with well-defined edges to the gel could be seen. In the ADA-GEL, rounded cells with the nucleus in the middle after printing can be found, while starting day 2, the cells began to spread out, and the cytoskeleton formed characteristic spindle-shaped extensions. This continued until day 14, the cell number increased and spread out like a 2D cell culture, while also penetrating the gel. Especially close to the surface, they formed an interconnected network within the hydrogel. Within the gel, colonies migrated and were smaller.

Quantification of the overview images of day 14 gave further insight on the growth pattern within the two gels (Fig. 5). In ADA-GEL, the surface was significantly more covered than in Alg/HA/Gel (92 ± 8% vs. 0%). Within the gel, colonies were smaller in ADA-GEL than in Alg/HA/Gel and covered a smaller area (10 ± 8% vs. 37 ± 8%). The surface in ADA-GEL was significantly more covered with cells than its inside where this was reversed in Alg/HA/Gel.

**Figure 6:**
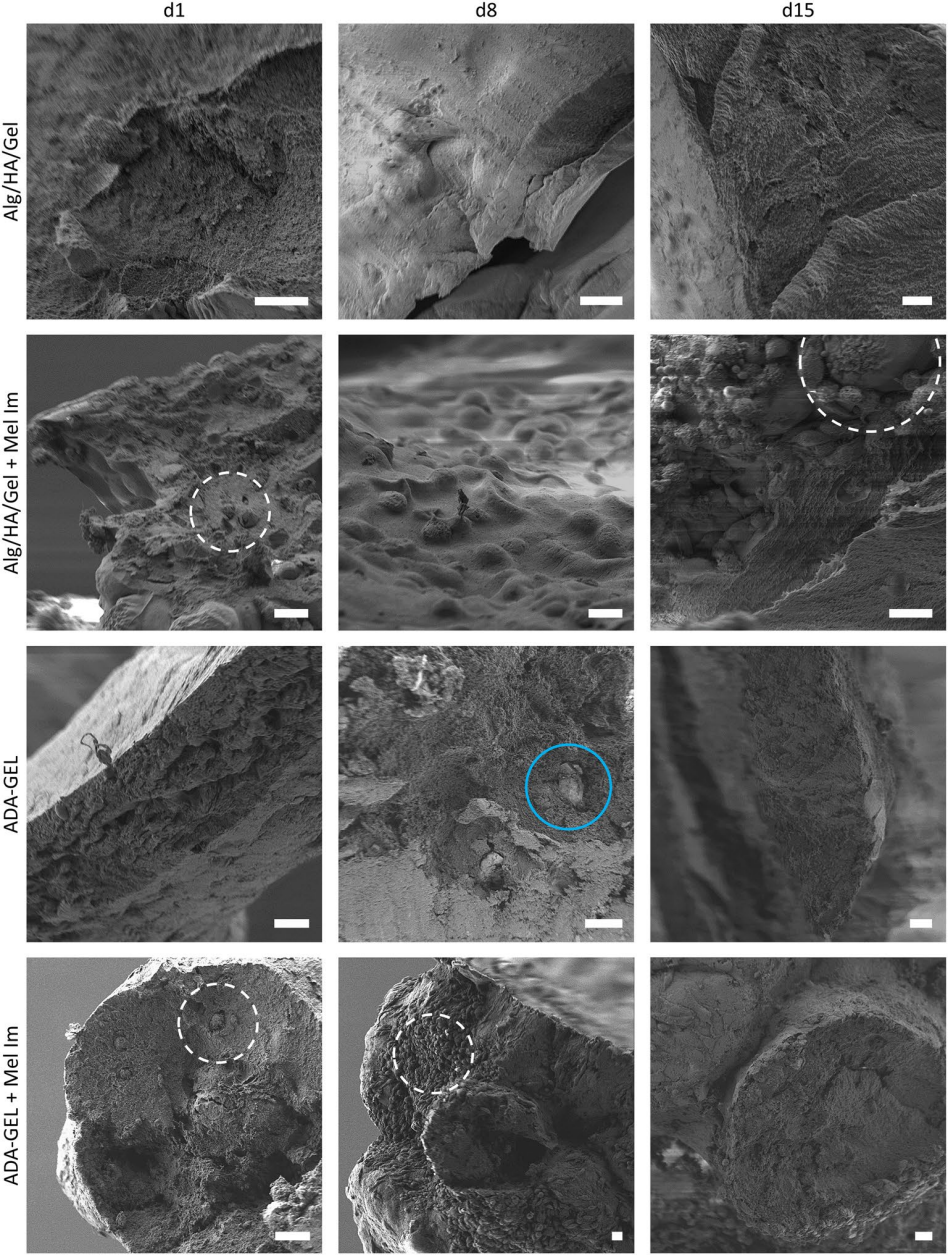
SEM Images of Alg/HA/Gel and ADA-GEL fractured grids with and without Mel Im cells over 15 days. SEM images of Alg/HA/Gel show a consistently fine structure of the ink. Mel Im cells appear as round colonies within the ink (marked on day 1 and day 15) encapsulated by a gel layer. In the ADA-GEL, particles can still be found trapped in the gel (marked in blue on day 8). The cells can also be seen within the bioink (marked) and also outside the gel (marked on day 8). Scale bar = 20 μm.

In the scanning electron microscopy images (Fig. 6), it appears that the Alg/HA/Gel has a similar structure to ADA-GEL on a cellular level. Breaking edges show a dense hydrogel structure. Over the incubation period of 15 days, this did not change for both inks. In ADA-GEL, trapped crystalline CaCO_3_ particles which did not completely dissolve during the pre-crosslinking, can still be found within the ink. Those can be found even after 15 days in culture. In Alg/HA/Gel, Mel Im cells can be found within the ink on all of the investigated days. Starting on day 8, the cells are found as colony spheres in the gel and covered by a layer of ink. On day 15, several individual cells can also be seen directly below and on the gel surface, and larger cell colonies can also be seen close to the surface. In the ADA-GEL, cells can be found in the gel in similar conditions. There are more cells on the gel surface on day 8 and day 15. The cells are not covered by a layer as they can migrate to the outside. The gels are covered with a dense cellular network.

**Figure 7:**
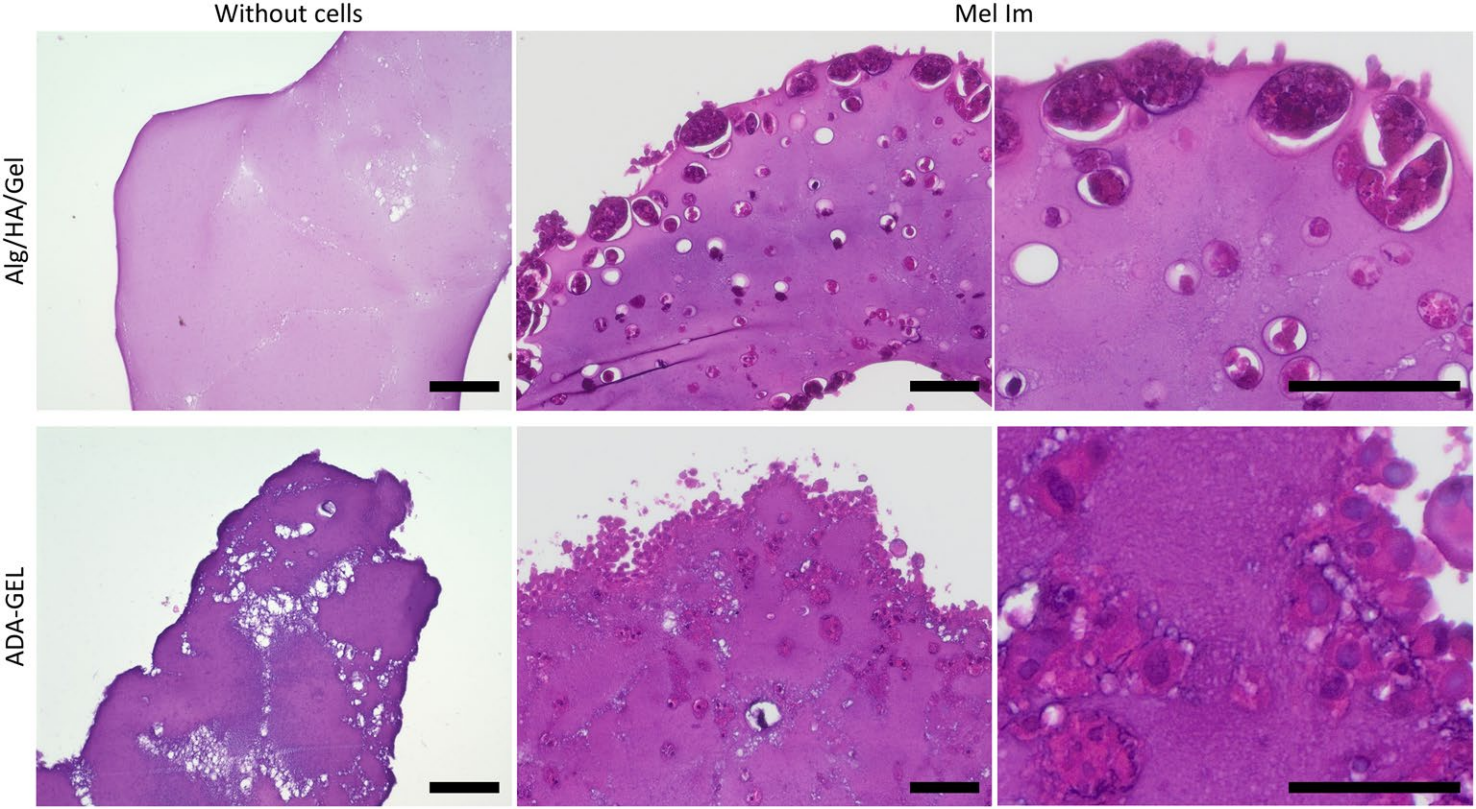
H&E stainings of Alg/HA/Gel and ADA-GEL with and without Mel Im cells on day 15. The H&E staining shows a smoother structure of Alg/HA/Gel, while ADA-GEL is perforated with pores. The cells in Alg/HA/Gel are located in sharply defined colonies within the gel, which become larger towards the edge of the gel; only a few cells can be found outside the gel. In contrast, the cell colonies in ADA-GEL are interconnected, and the cells also colonize the surface of the gel. Scale bar = 100 μm.

The histological analysis of the samples on day 15 (Fig. 7) showed various differences between the gels. The edges of the Alg/HA/Gel gels were smoother than those of the ADA-GEL gels without cells. The gel structure was also smoother. As with the SEM images, trapped CaCO_3_ particles could be found in the ADA-GEL, and bubbles could also be seen in some cases. When comparing the gels with cells, differences can be observed. In both gels, the cells preferred to grow at the interface with the cell culture medium but in Alg/HA/Gel, most of the cells were still inside the gel. They grew within the gel in smaller inclusions within small, individual, sharply defined colonies; the closer to the interface, the larger the colonies became, but they were still covered and enclosed by the gel (Spearman correlation coefficient r of -0.77). Colonies were also found in the ADA-GEL, but they were not as large. In the gel, the colonies were partially interconnected and formed networks. Closer to the interface, larger colonies were also found, but here, the gel was covered on the outside by cells that occupy the entire interface, often in multiple layers (r = 0.23).

## Discussion

In this study, we compared the two hydrogels Alg/HA/Gel and pre-crosslinked ADA-GEL with regard to the influence of melanoma cells on the gel and vice versa.

The cell line used here is Mel Im ^22^, isolated from cutaneous malignant melanoma of nodular type with a typical BRAF mutation ^23^. This type of melanoma is one of the most common and most aggressive ^24^. Given its origin form a metastasis, this cell line is well-suited for invasive research models.

Within this study, a printed approach was chosen instead of a cast one with more refined models in future in mind. Due to the printed approach, microporous structures different from cast models form; these effect cellular behavior ^25^. Advanced printed multi-component in vitro cancer research models may include blood ^26^ or even lymphatic vessels ^27^ to study melanoma metastasis and therapy.

As has already been shown, both gels offer good printability. In the filament fusion test carried out here, both hydrogels can be classified in the second-best category of printable inks. Another indicator is the diagonal crossing ratio (DCR), which indicates whether printed strands either retain their ideal shape or fuse together after stacking. In accordance with a previous study, ADA-GEL shows a DCR value closer to 1 than Alg/HA/Gel, which indicates better printability of the ink ^28,29^. This property of ADA-GEL was strongly influenced by the pre-crosslinking step which had a significant positive effect on the shape fidelity of the gel, while also shear thinning is given ^17^. The latter has a positive effect on cell survival. The negative effects of cellular deformation on the survival during extrusion-based printing can be further reduced by the incorporation of physiological calcium concentrations into the system ^30^. In this study, cylindrical needles were used for printability assays and conical needles for the cell-based assays. Better printability can be achieved using cylindrical needles compared to conical needles, although this also decreases cell viability ^31^.

Both gels provide RGD motifs due to the gelatin content which can serve as an anchor point for cells and thus contribute to improve cell viability ^32^. In the case of Alg/HA/Gel, in addition to the RGD motifs, there is also a CD44 interaction possibility via the containing HA. HA is known to act as a ligand of the CD44 receptor ^33^. This interaction is involved in processes such as cell growth and migration as well as angiogenesis ^34^. These are ideal conditions for using these inks in a 3D melanoma model.

The measured diameter shows that the Alg/HA/Gel ink without cells shrinks marginally less than ADA-GEL without cells over the measured period of 15 days (14% vs. 16%). The degradation of the gels was previously measured in terms of mass and shows comparable values for a period of 14 days. A weight reduction of 23.82% was observed for ADA-GEL and 34.87% for Alg/HA/Gel ^28^. It is possible that this is related to the fact that unbound gelatin in Alg/HA/Gel diffuses out of the gel over time. This effect is not as pronounced in ADA-GEL, as the gelatin is bound to alginate and the gel is further cross-linked using microbial transglutaminase (mTG). However, the data also show that ADA-GEL with cells behaves strikingly different, and the change from day 0 to day 1 in particular is significantly greater than that of Alg/HA/Gel with cells. The decrease in diameter over 15 days is also the most pronounced in this group. It can be concluded that this relates to the cross-linked gelatin. As mentioned previously, in the Alg/HA/Gel ink, the unbound gelatin can diffuse out of the gel, whereas the ADA-GEL is more firmly anchored in the gel and is, therefore, available to the cells for contraction and remodelling and thus, shrinks more than Alg/HA/Gel. It is known that invasive tumors have contracting forces on the surrounding gel ^35^, which could lead to further shrinkage.

The stiffness measurements reveal that all the gels have predominantly elastic properties and are in the range of physiological skin ^36^. Although there was a significant difference between the gels, the cells themselves did not have a significant effect on stiffness. This is contrary to experiments with decellularized tissue in which cells had a significant impact on the stiffness, making the tissue softer ^37^. Accordingly, a higher number of cells could increase the cellular influence. Comparing the storage modulus E′ measured in the rheometer with the Young’s modulus of MicroTester compression measurements of empty gels, similar ratios between Alg/HA/Gel und ADA-GEL can be observed ^28^. This is true even though in the present study gels were printed and not cast. Differences can be based on batch-to-batch variations as well as different processing routes (cast vs. printing), where properties of printed samples can alter based on printing parameters such as layer height and total infill. Similar ranges were also measured for ADA-GEL in nano-indentation experiments, indicating the reliability of the measurements in these studies even though different methods were used.

Furthermore, differences in cellular behavior can be seen in the gels. The cells can survive in both gels, though there are fundamental differences in the distribution in the gels. The cells in Alg/HA/Gel are closer to the surface and form larger colonies there, whereas they grow more extensively on the gel surface in the ADA-GEL. This can also be clearly seen from the diameter of the colonies and the distance to the edge of the gel. This is reflected in Alg/HA/gel via a correlation coefficient of -0.77. In ADA-GEL, on the contrary, no correlation can be determined in this respect (r = 0.23). In both gels, cell proliferation and survival is higher closer to the gel surface, as the nutrient exchange is expected to be more pronounced here and thus, the supply is better. In Alg/HA/Gel, the cells tend to grow in dense colonies within the gel. The cells cannot remodel the alginate backbone and, therefore, have more difficulty escaping the gel in favor of more space ^38^. Here, the alginate forms a network that cannot be remodeled. Colonies closer to the surface experience less compression force, hence the formation of larger colonies. In the ADA-GEL, the cells have the possibility of remodeling, as the oxidized alginate has bound the gelatin which can be degraded and thus, leaves flexible pores in the gel that allow cellular migration within the gel. This furthermore enables the cells to spread and have spindle-shaped morphology, as the bound gelatin serves as anchorage, an indicator that ADA-GEL mimics the tumor microenvironment better. Anchorage and spreading is not possible with standard unoxidized alginate which cannot be degraded by mammalian cells. Similar spreading has already been demonstrated in pre-crosslinked alginate with murine fibroblasts ^17^. In addition, migratory behavior of sprouting of endothelial cells was also shown in printed pre-crosslinked ADA-GEL, while in Alg/HA/Gel, it was not possible for the endothelial cells to form sprouts from spheroids, similar to Mel Im in this study ^28^. It has been demonstrated that the pre-crosslinking of alginate or ADA increases the stress relaxation amplitude of the hydrogel and facilitates migration, proliferation and spreading ^39^. Within the dense colonies, dead cells were also found in the present study. This can be explained by the fact that the agglomerated cells within a colony hinder the exchange of nutrients. This is further supported by the cytoskeleton staining which shows a very high cell density with dense interactions within the colonies.

SEM results support the data obtained so far. It is both compelling and simple to observe that the cells migrate out more quickly and also colonize the surface in ADA-GEL. In Alg/HA/Gel, cells are less likely to colonize the surface, as the cells cannot actively remodel the alginate backbone. With larger colonies, the cells may burst the surface and thus, reach the outside where they then further proliferate. Comparing SEM results of other experiments revealed a similar morphology and cellular behavior ^40^. However, it is not possible to quantify the SEM images because the gels behave differently from the cells during dehydration. This can be clearly seen in the SEM images of the cells in Alg/HA/Gel ink (day 8). Here, the cells are surrounded by the shrunken ink. This is a typical behavior of hydrogels, which change their characteristics during dehydration ^41^.

The histological data from day 15 again showed similar results. It was found that in ADA-GEL, the melanoma cells form multi-layered clusters on the outside or gradually replace the hydrogel. Here, MMP-2 presumably is the most important active MMP ^42^. The larger spherical colonies are clearly visible in Alg/HA/Gel. Some smaller colonies without cell nuclei, i. e. necrotic colonies, can be seen. It is typical for cancer that the tumor is necrotic inside, which can also indicate an aggressive type of cancer and the development of metastases in patients ^43^. Necrotic cells release their proteins, which is typical in tumor necrosis ^44^. The green and the red fluorescent proteins accumulate, resulting in super positioning, making differentiation of the FUCCI signal impossible.

## Conclusion

In conclusion, both bioinks proved to be sophisticated and versatile inks with satisfactory printability. The effect of the bioink chosen had a significant effect on the melanoma cell line while the effect of the cells onto the gels was less pronounced. Depending on the hypothesis and the model, both Alg/HA/Gel and pre-crosslinked ADA-GEL can be of use. Segregated colonies formed in Alg/HA/Gel while spreading, migration and remodeling was possible in ADA-GEL. Therefore, pre-crosslinked ADA-GEL was the more physiological hydrogel for mimicking the tumor microenvironment.

## Methods

### Cells and cell culture

In this study, the melanoma cell line Mel Im ^22^ was used as the experimental model. These cells were transduced with a FUCCI reporter for visualizing the cell cycle and positions ^45^. With the FUCCI indicator system, the nucleus of FUCCI-expressing cells fluoresces red during the G1 phase (Kusabira Orange 2, mKO2), yellow during the transition to the S phase (overlay of red and green), and green in the S phase, G2 phase and M phase (Geminin and Cdt1, respectively bound to Azami Green, mAG). Mel Im FUCCI cells were cultured in a culture medium composed of low glucose Dulbecco's Modified Eagle's medium (DMEM; Sigma-Aldrich St. Louis, MO, USA) with 10% fetal calf serum (FCS superior; Sigma-Aldrich) and 1% Penicillin–Streptomycin. The cells were incubated at 37 °C with 5% CO_2_.

### Bioink preparation

#### Alg/HA/Gel

In this study, the first bioink, Alg/HA/Gel, was created by combining alginate, hyaluronic acid, and gelatin, as established by Schmid et al*.* ^12^. The formulation included 0.5 wt% VIVAPHARM Alginate PH 176 (JRS PHARMA GmbH & Co. KG, Rosenberg, Germany), 0.1 wt% hyaluronic acid (1–2 MDa, CarboSynth Ltd, Compton, UK), and 3 wt% gelatin from porcine skin (gel strength ≈300 g Bloom, Type A, Sigma-Aldrich), all dissolved in phosphate-buffered saline (PBS, Sigma-Aldrich) in a beaker. The mixture was homogenized through stirring on a heated plate at 37°C for 1.5 h. To maintain the concentrations and prevent PBS evaporation, the beaker was covered with a sealing film.

#### ADA-GEL

ADA-GEL was prepared according to the protocol of Schulik et al. ^28^. In summary, oxidized alginate, ADA (with a 13% degree of oxidation) was dissolved in PBS at a concentration of 6.25 wt%. Furthermore, 6.25 wt% gelatin was also completely dissolved in PBS and mixed with 250 mM CaCO_3_ (Sigma-Aldrich). ADA and GEL with CaCO_3_ were homogeneously mixed in a 1:2 ratio. Directly before use, a 250 mM D-Glucono-δ-lactone (GDL, Sigma-Aldrich) solution was prepared and dropwise added to ADA-GEL and stirred over night at 37 °C to ensure thorough homogenization and proper consistency. The gel had a final concentration of 2.5% ADA, 2.5% GEL with 100 mM CaCO_3_, and 50 mM GDL.

#### 3D bioprinting

The bioinks were 3D printed using a Cellink INKREDIBLE + bioprinter (Cellink, Gothenburg, Sweden). To investigate the behavior of Mel Im FUCCI cells within the prepared bioinks, cells were suspended within with a cell density of 1 × 10^6^ cells ml^−1^. 37 °C warm ink was added to the cell pellet using a direct displacement pipette. This step was performed carefully to avoid subjecting the cells to excessive shear forces.

Once the ink and cells were adequately mixed, the 3-ml cartridges were prepared for the 3D bioprinter and stored in a bead bath at 37 °C until they were ready to be printed. Both bioinks exhibited temperature sensitivity due to the use of gelatin as a major component. The Alg/HA/Gel ink required cooling to 15 °C shortly ahead of printing to achieve the desired viscosity for optimal results. Hence, the cartridges were submerged in 15 °C cold water for 6 min briefly before printing. Conversely, the ADA-GEL bioink maintained a suitable viscosity at room temperature, due to the pre-crosslinked ADA. Therefore, the cartridges of this ink needed to reach room temperature before printing, which typically took 30–60 min.

Two different shapes were printed for the ink printing tests. First, a meander structure for the filament fusion test and a grid to measure the union of intersecting strands. Both structures were printed with a cylindrical nozzle with an inner diameter of 250 µm.

For the meander structure, parallel strands (length 20 mm) with a decreasing distance of 2 to 0.5 mm were printed. The smallest distance at where the strands do not yet run into each other was measured. 5 categories with increasing size can be distinguished: 0.5 mm (A), 0.75 mm (B), 1.0 mm (C), 1.5 mm (D) and 2.0 mm (E).

For the grid structure, 1 cm^2^ grids with two layers were printed and the diagonals of the crossed strands were measured and classified using the ideal diameter of crossed strands (354 µm with a tip with a diameter of 250 µm). The value of the optimal diagonal is divided by the measured diagonals. This results in the diagonal cross ratio (DCR), for which the values are better the closer they are to 1.

The printing process used a conical 0.41 mm diameter needle with printing pressure maintained between 15 and 25 kPa. For the rheological measurements and MPM analysis, solid round disks with a diameter of 20 mm and a height of 2 mm were printed. For SEM and histological analysis, 1 cm^2^ grids with three layers were printed. After printing, the samples were crosslinked: Alg/HA/Gel ink with a 0.1 M CaCl_2_ solution and ADA-GEL ink with a 0.1 M CaCl_2_ solution containing 5 wt% mTG (Ajinomoto, Tokyo, Japan). The samples were then immersed in the crosslinking solution for 30 min for disks and 10 min for grids, then washed with cell culture medium for two minutes. Subsequently, the samples were transferred to a 6-well plate with cell culture medium and placed in an incubator for further processing and analysis.

### Material characterization

Rheological tests were conducted to investigate the material behavior of the bioinks, specifically focusing on the storage and loss modulus with and without cells. The specimens were examined on day 1, 8, and 15 after printing. The disk-shaped samples were measured using a caliper on the corresponding days before performing the rheological analysis.

The tests were performed using a DHR3 rheometer from Texas Instruments, controlled by the TRIOS v. 5.5.22 software. A 20 mm 2-deg cone plate-plate compression fixture (TA Instruments, Eschborn, Germany) was used as the measuring geometry, and the experiments were conducted at a temperature of 37 °C in compression mode. During the measurements, each specimen was placed on the Peltier element of the rheometer and covered with cell culture medium to prevent drying. To establish a suitable amplitude at which the storage and loss modulus of the specimen remained constant, an amplitude sweep was initially performed at 1 s^−1^, ranging from 0.01 to 10% deformation using a calibration sample. This amplitude was crucial for the subsequent frequency sweep. A second calibration specimen was used for the frequency sweep, in which different preloads were tested. The preloads started at 0.25 N and increased by increments of 0.05 N until reaching a maximum of 0.6 N. The objective was to identify a preload where the axial force and plate distance remained constant throughout the measurement, indicating that the specimen could withstand the force without damage, while ensuring contact between sample and measurement geometry throughout the tests. Once an appropriate preload was determined, frequency sweeps were conducted on the remaining four specimens to investigate their material behavior. Technical replicates were measured at each point in time and their values averaged for the biological replicates.

### Cellular examination

#### Staining

For the multi-photon microscopy (MPM), the cytoskeleton was stained to visualize the cell morphology and interaction with the matrix. Here, samples were fixed on day 0, 7, and 14 (60 min with 3.75% paraformaldehyde in HBSS, Sigma-Aldrich and Thermo Fisher Scientific, Waltham, MA, USA, respectively). The cells were then permeabilized for 20 min (0.1% (w/v) TritonX-100 (Sigma-Aldrich), 5% (w/v) Sucrose (Merck, Darmstadt, Germany) dissolved in HBSS) and stained with Alexa Fluor 635 Phalloidin (Thermo Fisher Scientific; 8 µl ml^−1^ HBSS) for 1 h. Subsequently, the nucleus is stained with 4′,6-diamidine-2-phenylindole 1 µg ml^−1^ (DAPI, Thermo Fisher Scientific). The samples were stored in HBSS at 4 °C until the microscopic examination.

#### Multi-photon microscopy

MPM was employed in this study to observe and quantify the behavior of the cells within the samples. The position and cell cycle of the cells, as determined by fluorescence ubiquitination cell cycle indicator (FUCCI), were observed on different measurement days (days 1, 8, and 15). Additionally, the morphology of the cells or developing cell structures was examined on days 0, 7, and 14 after staining with Alexa Fluor 635 Phalloidin/DAPI.

A TrimScope II microscope (LaVision BioTec, Bielefeld, Germany) was used for multi-photon microscopy. The microscope utilized a Ti:Sa femtosecond pulsed LASER with a wavelength set to 810 nm for all experiments. The pulse frequency was 80 MHz, and the maximum LASER power was 1.2 W. The LASER beams were scanned using a beam scanner, collimated with a tube lens (infinity optics), and focused onto the specimen through an objective. A 10 × objective with a numerical aperture (NA) of 0.45 or a 25 × objective with an NA of 0.95 was selected depending on the specific application.

The fluorescence excited by the LASER was captured in the reflection direction through the excitation objective, and the signals were separated from the excitation light using a dichroic mirror. For the untreated samples where the FUCCI signal was to be isolated, a dichroic mirror with a reflection threshold of 560 nm was used. A similar dichroic mirror with a reflection threshold of 594 nm was used for the Alexa 635 Phalloidin/DAPI stained samples. The signals were subsequently filtered using bandpass filters and detected by photomultipliers (PMT). For the untreated samples, bandpass filters of 595/40 nm (PMT1) and 525/50 nm (PMT2) were used. For the stained samples, bandpass filters of 620/60 nm (PMT1) and 450/70 nm (PMT2) were used.

Microscopy of the samples was conducted by placing them in a sample tray and covering them with the respective medium. A coverslip was used when using the 10 × objective, while the 25 × objective, being a dipping objective, did not require a coverslip. Prior to microscopy, transmitted light was utilized to properly position and focus the sample under the objective. Once in position, the transmitted light was turned off, and multi-photon microscopy was performed.

The ImSpector Pro v.7_124 software (LaVision BioTec) was employed for system operation and image acquisition. For the analysis of non-treated samples, images were captured with a field of view measuring 1 × 1 × 1 mm^3^. Each voxel within the scanning microscopy image had a size of 4 μm^3^. This both ensured sufficient resolution for image processing while maintaining an acquisition time of approximately 5 min per image. Images were acquired from both the top and bottom sides of the specimen, providing additional comparative data. Seven images were randomly taken from the top, while three images were randomly taken from the bottom. The imaging process commenced from the surface of the specimen.

For the Alexa 635/DAPI staining morphology images, a field of view measuring 250 × 250 × 250 μm^3^ was utilized. In some instances, larger structures were recorded with a field of view of 500 × 500 × 500 μm^3^. Overview images for quantification were taken with a field of view of 1.024 × 1.024 × 1.024 µm^3^ Images were acquired at the highest possible resolution, resulting in a voxel size of 0.5 × 0.5 × 0.5 μm^3^.

The MPM images consisted, firstly, of a voxel on the surface of the sample in order to monitor the quantity and position of the cells in the sample. Secondly, it included detailed images of individual cells or cell clusters were taken in order to quantify the morphology of the cells within the two inks.

#### Scanning electron microscopy

To assess the changes in microstructure and the growth of the cells inside and on top of printed constructs, scanning electron microscopy (SEM, AURIGA®, Zeiss, Oberkochen, Germany) was conducted. To attain proper performance, samples of each ink with and without cells were incubated for 1, 8 and 15 days in medium. The grids were immersed in two fixation solutions (first consisting of: 0.1% glutaraldehyde, 667 mM paraformaldehyde, 146 mmol Sucrose; second consisting of: 0.3% glutaraldehyde, 1 M paraformaldehyde) for 1 h each to ensure proper fixation. Subsequently, the water content of the samples was replaced by ethanol trough an increasing ethanol series (30%, 50%, 60%, 70%, 80%, 90%, 95%, 99% ethanol, Carl Roth, Karlsruhe, Germany), with each step lasting at least 20 min. All solutions were supplemented with 4 mM CaCl_2_. Samples were then dried using a critical point dryer (EM CPF3000, Leica), cut to show the inside, fixed on carbon tapes and imaged.

### Histological staining

For histological staining, the samples were dehydrated according to the same protocol as described for SEM analysis. After dehydration, the samples were transferred to xylene (Carl Roth) and incubated for 20 min. Afterwards, the samples were embedded in paraffin wax (Carl Roth) and 3 μm cross-sections were cut with a microtome. The cross-sections were stained according to a standard protocol for H&E staining by the local Institute of Pathology, University Hospital Erlangen, and the pictures taken with a Keyence BZ-X810 microscope (KEYENCE, Osaka, Japan) with a 20× objective.

### Evaluation and statistical analysis

ImageJ 1.52p (NIH, Bethesda, Maryland, USA) was used for the quantitative analysis of microscopic images. Multiphoton-microscopy images were quantified by applying a threshold to the surface and the inside of z-projections of the gels. In H&E stainings, the colony diameters and their distances to the surface were measured manually in technical replicates. The statistical analysis was performed with GraphPad Prism Version 10.2.0 (GraphPad Software, San Diego, USA). Data was checked for statistical significance using a two- or three-way ANOVA followed by Fisher’s LSD test for multiple comparisons. Data is represented with means ± SD or individual values of biological triplicates. DCR is shown as box plot of technical replicates.

## Data Availability

Data available on request from the corresponding author.
